# Chemical–gene relation extraction using recursive neural network

**DOI:** 10.1093/database/bay060

**Published:** 2018-06-21

**Authors:** Sangrak Lim, Jaewoo Kang

**Affiliations:** Department of Computer Science and Engineering, Korea University, Anam-dong 5-ga, Seongbuk-gu, Seoul, South Korea

## Abstract

In this article, we describe our system for the CHEMPROT task of the BioCreative VI challenge. Although considerable research on the named entity recognition of genes and drugs has been conducted, there is limited research on extracting relationships between them. Extracting relations between chemical compounds and genes from the literature is an important element in pharmacological and clinical research. The CHEMPROT task of BioCreative VI aims to promote the development of text mining systems that can be used to automatically extract relationships between chemical compounds and genes. We tested three recursive neural network approaches to improve the performance of relation extraction. In the BioCreative VI challenge, we developed a tree-Long Short-Term Memory networks (tree-LSTM) model with several additional features including a position feature and a subtree containment feature, and we also applied an ensemble method. After the challenge, we applied additional pre-processing steps to the tree-LSTM model, and we tested the performance of another recursive neural network model called Stack-augmented Parser Interpreter Neural Network (SPINN). Our tree-LSTM model achieved an F-score of 58.53% in the BioCreative VI challenge. Our tree-LSTM model with additional pre-processing and the SPINN model obtained *F*-scores of 63.7 and 64.1%, respectively.

Database URL: https://github.com/arwhirang/recursive_chemprot

## Introduction

There is an increasing interest to find relationships between biological and chemical entities in the literature and store the relationship information in the form of a structured knowledgebase. An accurate and comprehensive knowledgebase can play an important role in many downstream applications in precision medicine. However, the gap between the information curated in the existing knowledgebases and the information available in the literature widens every day as the volume of biomedical literature rapidly increases. On average, >3000 papers are published daily in the biomedical domain alone. Although manual curation approaches are still widely used to ensure the quality of the contents in knowledgebases, completely manual approaches are too costly and cannot be scaled (1). An automated text mining-based relation extraction method can help speed up the curation process by automatically extracting relation candidates and providing them to human curators for verification.

The CHEMPROT track in BioCreative VI (2) aims to facilitate the development of text mining systems that automatically extract chemical–gene relationships from the literature. The organizers of the CHEMPROT track in BioCreative VI manually annotated chemical–gene entity relations in abstracts and divided the relations into 10 groups (http://www.biocreative.org/resources/corpora/chemprot-corpus-biocreative-vi/). Although all the groups are important in the biochemical and pharmacological perspective, only five groups were used for the evaluation. Table 1 shows the five groups and an example sentence for each group. The text given in the CHEMPROT challenge consisted of abstracts, entities and relations between two target entities. Since the main objective of this task was relation extraction, explicit named entity recognition was not required.

**Table 1.** Five groups of CHEMPROT relations to be used for evaluation

Groups	CHEMPROT relations	Sentence example
CPR:3	UPREGULATOR|ACTIVATOR|	<BC6ENT1>Amitriptyline</BC6ENT1>, but not any other tricyclic or selective serotonin reuptake inhibitor antidepressants, promotes <BC6ENT2>TrkA</BC6ENT2> autophosphorylation in primary neurons and induces neurite outgrowth in PC12 cells.
	INDIRECT_UPREGULATOR	
CPR:4	DOWNREGULATOR|INHIBITOR|	Ginseng total saponins, <BC6ENT1>ginsenosides Rb2, Rg1 and Rd</BC6ENT1> administered intraperitoneally attenuated the immobilization stress-induced increase in plasma <BC6ENT2>IL-6</BC6ENT2> level.
	INDIRECT_DOWNREGULATOR	
CPR:5	AGONIST|AGONIST-ACTIVATOR|	At 10(-6)M in transcription assays, none of these compounds showed progestin agonist activity, whereas <BC6ENT1>mifepristone</BC6ENT1> and its monodemethylated metabolite manifested slight <BC6ENT2>glucocorticoid</BC6ENT2> agonist activity.
	AGONIST-INHIBITOR	
CPR:6	ANTAGONIST	In another experiment, <BC6ENT1>cyanopindolol</BC6ENT1>, an antagonist of the <BC6ENT2>serotonin terminal autoreceptor</BC6ENT2>, also prolonged the clearance of 5-HT from the CA3 region.
CPR:9	SUBSTRATE|PRODUCT_OF|	Leukotriene A(4) hydrolase (<BC6ENT1>LTA(4)H</BC6ENT1>) is a cystolic enzyme that stereospecifically catalyzes the transformation of <BC6ENT2>LTA(4)</BC6ENT2> to LTB(4).
	SUBSTRATE_PRODUCT_OF	

The CPR:3 group was usually related to upregulation and words such as ‘activate’, ‘promote’ and ‘increase activity of’. The CPR:4 group was usually associated with downregulation and words such as ‘inhibitor’, ‘block’ and ‘decrease activity of’. The CPR:5 and CPR:6 groups were related to agonist and antagonist, respectively. Agonist and antagonist relationships are important for drug discovery and drug design. These four groups all have distinctive features. However, when multiple entities co-occur in a sentence, it is difficult to determine if a relationship exists between two target entities of interest. The CPR:9 group was related to substrate metabolic relations. Unlike the above four groups, the CPR:9 group did not have noticeable features, and thus the relations in this group were difficult to extract.

In the CHEMPROT challenge, almost all the relations occur between two entities in the same sentence. In the pre-processing step, we split an abstract into sentences and assumed a sentence with two entities to have a candidate relationship. In this challenge, most entities that have a relation are in the same sentence. Although relationships may exist between target entities in different sentences, we leave such cases for future work.

We built a relation extraction system for the CHEMPROT track in BioCreative VI using Recursive Neural Network based approaches. In this article, we introduce three Recursive Neural Network approaches that use the syntactical features of each node in a parse tree, exploiting the recursive structure of natural language sentences (3). The first approach uses tree- long short-term memory (LSTM) (4), a Recursive Neural Network model with dynamic batching. We implemented a position feature and a subtree containment feature to represent the locations of target entities. We used the first approach in the CHEMPROT challenge. After the CHEMPROT challenge, we conducted experiments using two additional approaches. The second approach uses the same model as the first approach but performs additional pre-processing. The third approach uses a new model called Stack-augmented Parser Interpreter Neural Network (SPINN) (5).

The original Recursive Neural Network is computationally inefficient mainly because its recursive tree parsing functions are incompatible with the batch operation. We use TensorFlow Fold (6) and SPINN to address this issue. TensorFlow Fold employs dynamic batching which adds another layer to enable batch operations, and SPINN uses a stack-based approach.

## Materials and methods

Relation extraction using text mining is a widely employed method in the biomedical field. The types of relation knowledge include protein-protein interaction (7, 8), mutation- (9) and chemical-disease (10) relations. In our previous work (11), we extracted drug–drug interactions (DDIs) using tree-LSTM with position and subtree containment features for a DDI task (12) that involved extracting four relations that can occur between two drugs. When compared with the DDI task, the CHEMPROT challenge was more difficult because it involved extracting five relations and typically had more numbers of entities in the text. However, the CHEMPROT challenge is similar to the DDI task in that it deals with the relationship between two entities. Thus, we used the same tree-LSTM model, which achieved competitive performance on the DDI task in the CHEMPROT challenge. After the challenge, we were able to improve the performance of the tree-LSTM model using the new pre-processing method. We also investigated the new recursive neural network model SPINN to further improve the performance.

**Figure 1.**
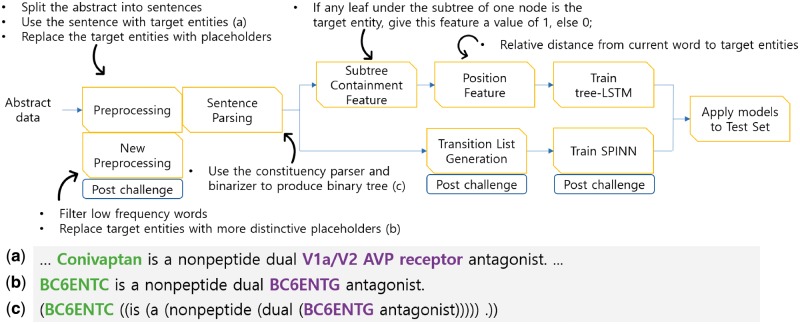
Overall system pipeline and data examples. We applied pre-processing and sentence parsing to the challenge data. The examples (a–c) are the input sentence, pre-processing, and parsing results, respectively. We also extract the subtree containment feature and the position feature for the tree-LSTM model and extract the transition list for the SPINN model. The steps labeled with ‘Post-challenge’ indicate the additional work done after the challenge to further enhance the performance.

The overall architecture of our system is presented in Figure 1. Each subcomponent of our tree-LSTM based model is explained in subsequent sections. The tree-LSTM model with additional pre-processing and the new SPINN model are discussed in the Post-Challenge Enhancements section.

### Pre-processing

Pre-processing involves sentence splitting and anonymizing target entities and chemical compounds. Abstract data usually consists of several sentences. However, we found that almost all the gold-standard relations exist between two target entities in the same sentence. Therefore, we split an abstract into sentences and assumed that a sentence had a candidate relationship if it contained at least two entities. We used only the sentences with candidate relationships and ignored the other sentences. Among the candidate relationships, we labeled the gold-standard relations as true instances and the others as negative instances. An example of a true instance is provided in Figure 1a.

Biochemical entities usually have long and complex names. For simplicity, we replaced the names of target entities with placeholders such as ‘BC6ENT1’, ‘BC6ENT2’ and so on. Also, we employed the ChemDataExtractor (http://chemdataextractor.org/download) to find chemical entities, and we replaced the names of the chemical entities with ‘CHEM’ (13). An example of this anonymization process is given in Figure 1b.

### Parsing sentences

Recursive Neural Network models use the syntactical features of each node in a constituency parse tree. We used the Stanford NLP library (14) to transform a sentence into a constituency parse tree. After the parsing process, we used the ‘binarizer’ provided by the Stanford Parser to convert the constituency parse tree into a binary tree. A binary tree is provided in Figure 1c.

### Subtree containment feature generation

We calculated the subtree containment feature in the parsing stage. The subtree containment feature indicates that a certain subtree contains an important entity. When one of the target entities exists in the leaves of the current node, the subtree containment feature is given a value of one; otherwise, it is given a value of zero.

During the training process, we consider the effect of each feature through the vector representation of features. Since the dimension size of the word embedding is larger than 100, it is not desirable to represent the subtree containment feature using only one-digit feature. The effect of one-digit feature can be dominated by features with higher dimensions such as word embedding feature. To avoid this problem, we converted the subtree containment feature into a vector with a size of 10 in the tree-LSTM model. We decided the size of the feature vector by a hyperparameter search process described in Table 4. If the value is one, every element of the vector is one; otherwise, every element in the vector is zero. This subtree containment feature is not used for the SPINN model. A more detailed explanation about this feature generation process is given in the Supplementary Material.

### Position feature generation

**Figure 2.**
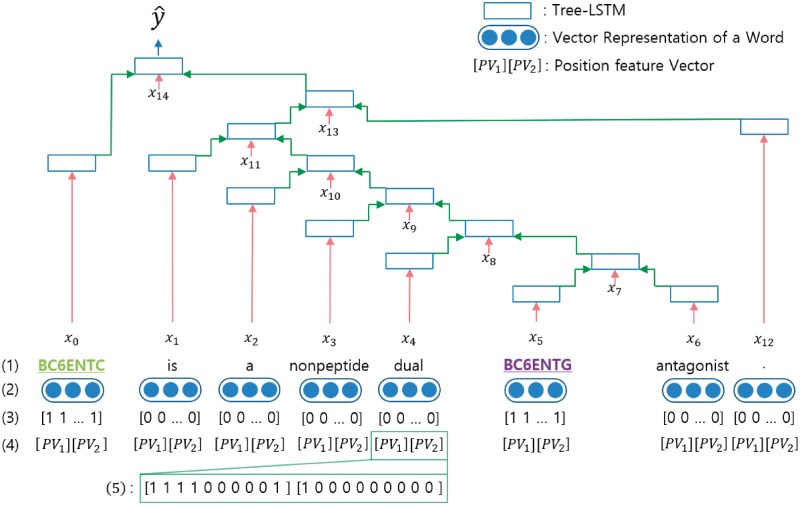
The architecture of our tree-LSTM model. (1) The target words in the sentences are underlined. (2) Vector representations of words from a pre-trained word embedding. (3) Vector form of subtree containment feature for each leaf. (4) Position feature vectors. 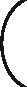
PV
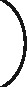
_1 and 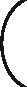
PV
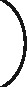
_2 are the relative distances of the first and second target words from the current word, respectively. (5) An example of the position feature vector when the current word is ‘dual’.

Position feature embedding represents the relative distance from each word position in a sentence to target entities (15). Every word in a sentence has two relative distances [$d_{1}$, $d_{2}$], where $d_{i}$ is the relative distance from the current word to i th target entity. For example, in Figure 2, the word ‘dual’ is the fourth word that follows the first target entity and it is located right before the second target entity. Therefore, the node of the word ‘dual’ has the position feature of [4, −1].

In the training phase, each relative distance is converted into a vector with a size of 10 for the same reason that the subtree containment feature is converted into a size-10 vector. Table 2 shows the vector representation based on the relative distances. Note that when the distance difference is 5 or less, the vector is assigned to each difference value. If the distance is >5, the same vector is given in units of 5. We skip the columns ranging from −5 to −∞ of the relative distance due to space limitations. Since there are two distances, the total vector size of the position feature embedding is 20. This position feature is not used for the model SPINN. A more detailed explanation on this feature generation process is given in the Supplementary Material.

**Table 2.** Vector representation according to the distance between one of the target entities and the current word

Relative distance	−5	−4	−3	−2	−1	0	1	2	3	4	5	6–10	11–15	16–20	21–∞
	0	0	0	0	0	0	1	1	1	1	1	1	1	1	1
	0	0	0	0	0	0	0	0	0	0	0	0	0	0	1
	0	0	0	0	0	0	0	0	0	0	0	0	0	1	1
	0	0	0	0	0	0	0	0	0	0	0	0	1	1	1
	0	0	0	0	0	0	0	0	0	0	0	1	1	1	1
	1	0	0	0	0	0	0	0	0	0	1	1	1	1	1
	1	1	0	0	0	0	0	0	0	1	1	1	1	1	1
	1	1	1	0	0	0	0	0	1	1	1	1	1	1	1
	1	1	1	1	0	0	0	1	1	1	1	1	1	1	1
	1	1	1	1	1	0	1	1	1	1	1	1	1	1	1

### Word embedding

Word embedding is a set of vectors that are trained by an unsupervised language model. Using word embedding with a neural network is a widely employed method for improving NLP performance (16, 17). We used the PubMed-and-PMC-w2v word embedding, which was obtained from published materials (http://evexdb.org/pmresources/vec-space-models/) (18). The source data of the word embedding was collected from biomedical texts, and 229 810 015 source sentences were used for the word embedding. We also tested other word embeddings with different dimensions obtained from different sources, but this word embedding performed the best because it was trained on the largest amount of data. The word embedding is initialized using the gensim Word2Vec library (19). The dimension size of the word embedding is 200.

### Recursive neural network with tree-LSTM

LSTM is a popular variation of the recurrent neural network (20). General LSTM is used for sequential data such as sentences. We implemented tree-LSTM to apply the LSTM architecture to our tree-structured data (4). A node in tree-LSTM receives input from two children nodes and updates the hidden state of the current node using the input. The architecture of our tree-LSTM model is presented in Figure 2.

The input of tree-LSTM for a node is always the node's word and the state values (memory cell and hidden state) of the two children nodes. After receiving a parse tree to train our model, we look up the pre-trained word embedding to assign real-valued vectors to each word. If a node is not a leaf and does not have an associated word, the word vector is filled with zero. If a node is a leaf and does not have children nodes, state values are filled with zero. Our model is based on the recursive neural network architecture of the child sum tree-LSTM model (4).

Let $x_{j}$ denote the concatenation result of the vector representation of a word with feature vectors. For any node *j*, we have two forget gates (one for each child) and denote the sub-node expression of the forget gates for *k*th child as $f_{jk}$. The *B*(*j*) is the set of values (including $h_{k}$ and $c_{k}$) from the children of node *j*, and the size of *B*(*j*) is 2 since we use a binary tree. Also, *i*, *f*, *o*, *c* and *h* are the input gate, forget gate, output gate, memory cell and the hidden state, respectively. In the expression of each gate, $e_{j}$ is the result vector of tracking LSTM which is required for SPINN. Since the tree-LSTM model does not use this vector, the vector is filled with zero for tree-LSTM model. $u_{j}\;$is a temporary vector used in the computation of the memory cell state, and drop(x) is a recurrent dropout function (21). Recurrent dropout improves performance by minimizing memory loss which is common when dropout is applied to a recurrent neural network. The mask is a sampled vector from the Bernoulli distribution with the success probability keep_p. Our tree-LSTM equations are described below.

(1) $$\tilde{h_{j}}=\;\sum_{k\in B\left(j\right)}h_{k},$$

(2) $$i_{j}=\;\sigma (W^{i}\left[x_{j},\tilde{h_{j}},e_{j}\right]+\;b^{i})$$

(3) $$f_{jk}=\;\sigma (W^{f}\left[x_{j},h_{k},e_{j}\right]+\;b^{f})$$

(4) $$o_{j}=\;\sigma (W^{o}\left[x_{j},\tilde{h_{j}},e_{j}\right]+\;b^{o})$$

(5) $$u_{j}=\;tanh(W^{u}\left[x_{j},\tilde{h_{j}},e_{j}\;\right]+\;b^{u})$$

(6) $$c_{j}=\;i_{j}⊙drop(u_{j})+\;\sum_{k\in B(j)}f_{jk}⊙c_{k}$$

(7) $$h_{j}=\;o_{j}⊙tanh(c_{j})$$

(8) $$drop\left(x\right)=\;\left\{\begin{array}{cc} mask*x,\; & if\;train\;phase,\\ x\; & otherwise \end{array}\right.$$

Equations (9) and (10) represent the fully connected layer we use as the output layer. The fully connected layer output size is the number of groups (one false group and five classification groups). At each node *j*, we choose the predicted label $\hat{y_{j}}$ for a given output. However, since the predicted value of the internal nodes in the tree is not important, we take only the predicted values from the root node of the entire sentence when the final score is calculated. We use the softmax cross-entropy classifier to calculate the cost function, *m* is the total number of items in the training set.

(9) $$\tilde{p}\left(y||x_{j}\right)=\;W^{{(f}_{c})}h_{j}+\;b^{{(f}_{c})}$$

(10) $$\tilde{y}=\;argmax\;\tilde{p}\left(y||x_{j}\right)$$

(11) $$J\left(\theta \right)=-\frac{1}{m}\sum_{k}^{m}y^{k}log(softmax\left(\tilde{p}\left(y^{k}||x^{k}\right)\right))$$

We use the Adam optimizer for gradient descent optimization. The input vector of a node in a tree uses the subtree containment feature vector, the position feature vector and the vector representation of a word in a sentence. The size of the whole input vector $x_{j}$ is 230 (10 + 20 + 200).

The original tree-LSTM model (4) used l2 regularization. We implemented our tree-LSTM model using recurrent dropout (21) instead of l2 regularization and found that recurrent dropout is equally effective.

### Ensemble method

Random weight initialization typically affects the results of neural networks. In the CHEMPROT challenge, it is difficult to build a robust neural network model that can produce consistent results. The prediction results of ambiguous instances can vary depending on how the model is trained. We resolve this problem to some extent by reducing the variance of our model using the ensemble method (22). We sum the output probabilities (logits) of ensemble members, which are generated using the same neural network model with random weight initialization. Our tree-LSTM model used in the BioCreative VI challenge employs six ensemble members. The two post-challenge enhancements use 10 ensemble members. A more detailed explanation on the ensemble process is given in Supplementary Material.

### Implementation detail

We use TensorFlow to implement our three approaches (23). Most deep learning libraries such as TensorFlow assume that machine learning models are static, which makes it difficult to use them for dynamic structure models such as Recursive Neural Network. We implement our tree-LSTM model using TensorFlow Fold which provides dynamic batching to solve the dynamic structure problem (6). A node in a parsed tree has its own tree-LSTM operation. Given parsed trees of diverse topologies, the dynamic batching algorithm re-groups operations of the same depth in a tree together. The re-grouped operations can be easily batched for efficient computation. In fact, Looks *et al.* (6) showed that in some cases dynamic batching can be up to 120 times faster than manual batching.

## Post-challenge enhancements

### Additional pre-processing

During the CHEMPROT challenge, we anonymized the target entities with ‘BC6ENT1’, ‘BC6ENT2’ and so on. Such sequential anonymization is good for generalization, but this CHEMPROT challenge was focused on how chemical entities affect gene entities. After the challenge, we applied a different anonymization strategy; we replaced the chemical entity with ‘BC6ENTC’ and the target gene entity with ‘BC6ENTG’. In addition, any entities other than the target entities were replaced with ‘BC6OTHER’.

When we looked up the embedding word vector, we assigned a random vector to a word that did not exist in the pre-trained embedding. Some words appear only once or twice in the whole dataset, which may act as noise. To reduce noise in the dataset, we filtered words that appeared less than three times. The filtered word vectors were initialized using a fixed random vector. We also applied this additional pre-processing method to SPINN.

### SPINN with additional pre-processing

Bowman *et al.* (5) introduced a new recursive neural network model called SPINN. The SPINN model performed well on the Stanford Natural Language Inference corpus which is used to determine whether two sentences correspond or contradict. With the help of the shift-reduce parser, the SPINN model uses the sequential structure instead of the recursive data structure.

**Figure 3.**
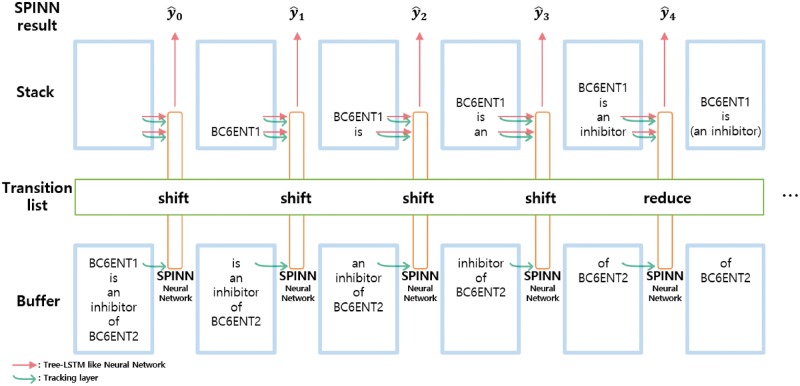
The SPINN model which implements a shift-reduce parser for each transition step.

The shift-reduce parser uses the following three data structures: a buffer that contains the words of a sentence, a stack that contains incomplete trees, and a transition list that contains a transition operation at each timestep. The initial state of the buffer is the list of words in a sentence, and the initial state of the stack is an empty list. For every timestep, the parser retrieves one of the following two transition functions from the transition list: shift and reduce. The shift function moves the top word of the buffer to the stack. The reduce function merges the top two nodes from the stack, and the pair of nodes become a subtree. Figure 3 describes the shift-reduce parser process.

We explain the transition list generation process of the SPINN model. Given a set of parsed trees, we make the buffers and the transition lists using the trees. When a word appears, we add the shift function to the transition list, and when the right parenthesis appears, we add the reduction function to the transition list. These lists of data are sequential, and the batch operations can be applied to the list for efficient computation. A node in a tree is enclosed in parentheses as shown in the following example:

**Table:** 

**Parsed Tree:**	(BC6ENT1 (is (an inhibitor) (of BC6ENT2)))
**Buffer:**	[BC6ENT1, is, an, inhibitor, of, BC6ENT2]
**Transition list:**	[shift, shift, shift, shift, reduce, shift, shift, reduce, reduce, reduce]

The SPINN model implements the shift-reduce parser to make a tree at each transition step, using the buffer and the transition list. The main neural network layer of SPINN is similar to that of our tree-LSTM model. The input of the main network layer of SPINN is the top two nodes of the stack. Also, SPINN has the tracking layer which is a simple LSTM layer whose inputs are the top element of the buffer and the top two elements of the stack. The authors of the SPINN model stated that the tracking layer contains supplementary feature information. The result of the tracking layer is fed to the main tree-LSTM algorithm and it becomes the $e_{j}$ in Equations (2–5). Since the tracking layer is a simple LSTM layer, we skip the equations for the tracking layer.

We do not apply the position or subtree containment features to the SPINN model because it is difficult to implement the features in a sequential manner.

## Results

### Data corpus

The CHEMPROT challenge organizers used the PubMed abstracts published between 2005 and 2014 as the CHEMPROT challenge data. Domain experts manually annotated the corpus following the strict guidelines of the organizers. After we pre-processed the given data, the number of negative instances was more than three times larger than the number of positive instances. Table 3 shows the statistics of the pre-processed corpus. We filtered several instances during the pre-processing. There are 14 instances in the training set where target entities are overlapped. There are 10 confusing instances which have two or more relation groups with the same target entities. We found that the test and the development sets also have confusing instances. If there were many confusing instances in the training set, we would have to include the instances in the training phase. However, since only 10 instances exist, we filtered them to reduce noise.

**Table 3.** The statistics of the BioCreative VI CHEMPROT corpus after pre-processing

Dataset	Abstract	Positive	Negative	Ratio
Train_orig	1020	4157	16 964	1:3.08
Develop_orig	612	2, 416	10, 614	1:3.39
Test_orig	3399		58, 523^a^	
Train	1020	4133	16, 522	1:2.99
Devel	612	2412	10 362	1:3.29
Test	3399	3444	10 999	1:3.19

The first three datasets are the original datasets used during the challenge.

a The challenge organizers appended dummy data to the test set to prevent from manual annotation by participants.

For the CHEMPROT challenge, the organizers appended dummy data to the test set to prevent participants from manually annotating. We combined the training and development sets, and trained our model on the combined set for the final evaluation.

### Hyperparameter

Training in machine learning involves adjusting values in weight vectors for a specific task. Apart from the weight vectors, almost all machine learning models have hyperparameters that determine how the models’ training should work. For example, the hidden unit size is one of the hyperparameters in deep learning models. The hidden unit size refers to the number of nodes of a hidden layer between the input and output layers. Generally, as the hidden unit size increases, a trained model can handle more complicated tasks. But if the hidden unit size is too large, the trained model can overfit the training samples, which makes it difficult to apply the model to new data. When testing a set of hyperparameters, we train the model on the training set with specific hyperparameter values, and evaluate the model’s performance on the validation set. To find the optimal hyperparameters, we conducted two rounds of experiments. The first round of experiments uses a randomized approach. Because the search space consisting of all the possible combinations of parameter values is too large, we repeated the random selection of parameter value combinations, and trained and validated the model. In the first round, we chose the best performing parameter values. Since the random selection of values is the sparse approximation of the optimal values, we need to perform more in-depth experiments. We then conducted another round of experiments to find the optimal parameters within the small search space near the hyperparameters found in the first round. A test unit is a measurement unit used for the hyperparameter search process. Table 4 provides the test ranges and test units of the hyperparameter search process. Because the CHEMPROT challenge provides both the training set and the development set, we tested the hyperparameters on the development set, while our models were trained on the training set. We also found the optimal hyperparameters for the SPINN model; the other hyperparameters were set to default values.

**Table 4.** Process for finding the best hyperparameter

Model	Parameter	Test range	Test unit	Selected
TreeLSTM	Batch size	64–512	64	256
	Hidden unit size	64–512	64	256
	Learning rate	0.0005–0.01	0.0005	0.001
	Keep probability	0.5–0.9	0.1	0.5
	Subtree containment^a^	2–10	2	10
	Epoch	500–1000	100	1000
SPINN	Batch size	64–256	64	256
	Hidden unit size	64–256	64	256
	MLP dropout	0.5–0.9	0.1	0.5

MLP, Multi-Layer Perceptron.

a Subtree Containment Size.

### Performance

We report the average of the five repeated results of the single models. The performance of the ensemble method is shown as well. The experimental results on the test set are shown in Table 5. In the CHEMPROT challenge, although each team could submit up to five results, we report only the top result of each team. A total of 13 teams participated and our team placed fourth in the CHEMPROT challenge. The micro-averaged F1-score of our tree-LSTM model was 58.5%. We report the scores of the other two enhancements below.

**Table 5.** Comparison between the results of our Recursive Neural Network systems and other top three CHEMPROT challenge results

Rank/Team ID (model)		P (%)	R (%)	F (%)
Challenge results				
1	TEAM_430	**72.6**	57.3	**64.1**
2	TEAM_403	56.1	**67.8**	61.4
3	TEAM_417	66.0	56.6	60.9
4	Our Tree-LSTM (ensemble)	67.0	51.9	58.5

Our Post-Challenge Enhancements				
	Tree-LSTM (single) +pp	65.7	58.1	61.7
	Tree-LSTM (ensemble)+pp	70.0	58.4	63.7
	SPINN model (single)+pp	61.5	**58.9**	60.2
	SPINN model (ensemble)+pp	**74.8**	56.0	**64.1**

Note: P, R and F denote Precision, Recall and F1 score, respectively; pp denotes the new pre-processing method applied to only the post-challenge models; (ensemble) is the weighted voting of 10 instances of the same models that are independently trained with randomly initialized weights; (single) represents the result of a single model instance.

After the CHEMPROT challenge, challenge organizers provided the challenge results along with the evaluation script. The evaluation script automatically computes the micro-averaged *F*-score of a given prediction on the gold-standard dataset. The SPINN single model and the SPINN model with the ensemble method achieved *F*-scores of 60.2 and 64.1%, respectively. Our tree-LSTM single model with additional pre-processing and our model with the ensemble method achieved *F*-scores of 61.7 and 63.7%, respectively. Our enhanced models using additional pre-processing methods outperform the first tree-LSTM model. Our tree-LSTM single model achieves better performance than the SPINN single model because the position and subtree containment features of the tree-LSTM model are helpful in locating target entities. The performance of the SPINN model with ensemble method exceeds that of the tree-LSTM model with ensemble method. It seems that the additional tracking layer is helpful. The SPINN model yields fewer false positives and achieves higher precision.

Several researchers combined two or more different machine learning models to improve performance in biomedical relation extraction (24–26). For example, Zhou *et al.* (24) utilized a linear combination of a feature-based model, a kernel-based model and a neural network model. Although it is difficult to achieve, we improved performance using only one recursive neural network model. Our model can be integrated with other models to further improve performance.

## Discussion

### Error analysis

The most common type of error is predicting a gold-standard class as a ‘False’ class. The confusion matrix of the SPINN model (ensemble) obtained on the test set is shown in Table 6.

**Table 6.** Confusion matrix for the SPINN model (ensemble) result on the test set

Gold	False	CPR:3	CPR:4	CPR:5	CPR:6	CPR:9
Pred^a^						
False	10 410	281	566	72	95	396
CPR:3	134	331	26	1	0	4
CPR:4	266	51	1064	0	4	6
CPR:5	16	0	0	107	4	0
CPR:6	30	0	1	3	189	0
CPR:9	100	1	1	0	0	238

a Pred is the prediction result.

Before describing error cases, we explain how all our models make a candidate relation, which may help in understanding the process of finding a relation. There are several entities in a sentence and we select one target gene and one chemical to make a candidate relation. Any entity that we do not select as a target entity can be a target entity in another candidate relation. We considered all the possible target gene–chemical pairs as candidate relations. For example, the first row in Table 7 shows the candidate relation between ‘zinc’ and ‘histone deacetylase’. The candidate relation between ‘zinc’ and ‘HDAC’ is also possible.

**Table 7.** Types of errors and corresponding example sentences from our SPINN model

Predicted results	A representative example sentence
Answer: CPR:4 (INHIBITOR) Predicted: -	Small molecules bearing hydroxamic acid as the <BC6ENTC>**zinc**</BC6ENTC> binding group have been the most effective <BC6ENTG>**histone deacetylase**</BC6ENTG> inhibitors (<BC6OTHER>HDAC</BC6OTHER> i) to date.
Answer: CPR:3 (ACTIVATOR) Predicted: -	<BC6ENTC>**CPT-11**</BC6ENTC> and <BC6OTHER>SN-38</BC6OTHER> may also stimulate the production of pro-inflammatory <BC6ENTG>**cytokines**</BC6ENTG> and <BC6OTHER>prostaglandins</BC6OTHER> (<BC6OTHER>PGs</BC6OTHER>), thus inducing the secretion of <BC6OTHER>Na(+)</BC6OTHER> and <BC6OTHER>Cl(-) </BC6OTHER>.
Answer: CPR:4 (INHIBITOR) Predicted: CPR:3 (ACTIVATOR)	<BC6ENTC>**Dimemorfan**</BC6ENTC> pre-treatment also attenuated the KA-induced increases in <BC6ENTG>**c-fos**</BC6ENTG>/c-jun expression, activator protein-1 DNA-binding activity, and loss of cells in the CA1 and CA3 fields of the hippocampus.

In the Introduction section, we list the characteristic words of each relation, but even if a characteristic word in the list appears, it may not be directly related to the relationship between two target entities. We analyzed the error cases of the SPINN model with the ensemble method. Below, we introduce three representative error cases:

1. Failure to understand sentence structure
  As shown in the first row of Table 7, the chemical ‘zinc’ is an inhibitor of the target gene ‘histone deacetylase’. Our model predicted the relation as ‘False’ because our model could not understand the sentence structure. On the other hand, our model correctly predicted the other relation ‘zinc-HDAC’ in the sentence. It seems that our model did not properly learn how the word ‘inhibitors’ represents the ‘CPR:4’ group in certain sentence structures. To solve this error type, more training instances are required.
2. Failure to detect coordination
  Coordination relation is expressed in various forms in sentences, and we have identified cases where our model does not properly detect coordination. Most of the time, coordination relation is expressed with a comma, parenthesis or special words such as ‘and’, ‘or’. In the second row of Table 7, the chemical ‘CPT-11’ and the word ‘SN-38’ are both equally emphasized. Since every possible chemical–gene candidate relation is given to our model, ‘SN-38’ becomes a chemical for another instance. Our model correctly extracted the relation ‘SN-38–cytokines’. However, our model incorrectly predicted the second row of Table 7 as ‘False’ because it could not find the information indicating that the chemical ‘CPT-11’ has coordination relation with ‘SN-38’. An independent module that searches for equally emphasized words may help prevent this type of error.
3. Misclassifying inhibition as activation and vice versa.
  As shown in Table 6, there are 26 cases where our model predicts ‘CPR:4’ instances as ‘CPR:3’ instances. Our model predicts the third row of Table 7 as ‘CPR:3’ largely because of the word ‘increases’. However, a human reader can see that ‘target gene expression’ is attenuated by the chemical and the word ‘increases’ is not related to the target entities. More training data would help address this type of error.
  In the case of the relation groups ‘CPR:5’ and ‘CPR:6’, our model correctly predicts the relations most of the time if our model does not predict the relation group to be ‘False’. However, our model confuses the relation groups ‘CPR:3’ and ‘CPR:4’, even if it does not predict the relation as ‘False’. Our model confuses these groups because the representative words of the ‘CPR:3’ and ‘CPR:4’ groups are general words. While the representative words of the ‘CPR:5’ and ‘CPR:6’ groups consist of a small number of specific words. For example, group ‘CPR:3’ instances occur with the words related to ‘ACTIVATOR’, such as ‘increase’, ‘promote’ or ‘activate’, all of which are common words. On the other hand, group ‘CPR:5’ instances occur with the words related to ‘AGONIST’, such as ‘agonist’, which are uncommon.

### Summary and future directions

To summarize, we have looked at three cases where our model does not understand the structure of a sentence, or the function of a coordinating conjunction, and does not distinguish different class features. To address these problems, we plan to improve our models by using more training data, and by checking for coordinate conjunctions.

In addition, although it is not considered in this challenge, it is important to extract cross-sentence relations in biomedical literature. Several studies (27, 28) introduced a framework for cross-sentence relation extraction using a dependency tree graph on several sentences. The main approach is to add a link between two root nodes of adjacent sentences. The root node is the top node of a dependency parsed sentence. This approach was later adopted by Peng *et al.* (29). They also proposed graph LSTM networks. Since our recursive neural network models are based on a parsed tree structure, we believe that this approach can also be applied to our models to tackle the cross-sentence extraction issue. We will further investigate and address this issue in our future work.

## Conclusion

The CHEMPROT track in the BioCreative VI challenge offered the valuable opportunity to improve chemical–gene text mining. We implemented Recursive Neural Network architectures to extract chemical–gene relationships from sentences in natural language. We showed that simple recursive neural network-based models can achieve performance comparable to that of more complex models. For future work, we plan to extract relations in abstracts

The source codes of our Recursive Neural Network models are freely available at: https://github.com/arwhirang/recursive_chemprot

## Supplementary data

Supplementary data are available at *Database* Online.

*Conflict of interest*. None declared.

## Funding

This research was supported by the National Research Foundation of Korea (http://www.nrf.re.kr/) grants (NRF-2016M3A9A7916996, NRF-2017M3C4A7065887, NRF-2017R1A2A1A17069645 to JK). The funders had no role in study design, data collection and analysis, decision to publish, or preparation of the manuscript.

## Supplementary Material

Supplementary Data
