# Supplementary material for: Chemical–gene relation extraction using recursive neural network
Source: Database (Oxford). 2018 Jun 21;2018:bay060. doi: 10.1093/database/bay060 (PMC6014134; doi:10.1093/database/bay060)
Supplement: Supplementary Data [file bay060_supp.docx]

Supplementary Material

We illustrated our tree-LSTM model in Figure 1 in the manuscript. We focused on explaining each process, but our explanation could be enhanced with examples. In this Supplementary Material, we will explain how each feature and tree-LSTM values are transferred from one node to the other using examples.

**Subtree containment and position feature generation process explained with example.**

We describe here the subtree containment and position feature processes with examples.


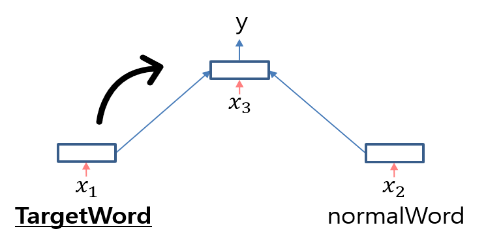
Subtree Containment Feature Generation

Our reasoning behind the subtree containment feature is simple. We want to put more focus on target words. In the leaves, we assign the subtree containment (SC) feature a value of 1, only if the word is a target word. In the tree structure, we have parent and child nodes. When one of the child nodes contains the target word, we transfer the SC feature to the parent node. This is illustrated in Supplementary Figure 1.

Supplementary Figure 1 The SC feature of target word node is transferred to its parent node.


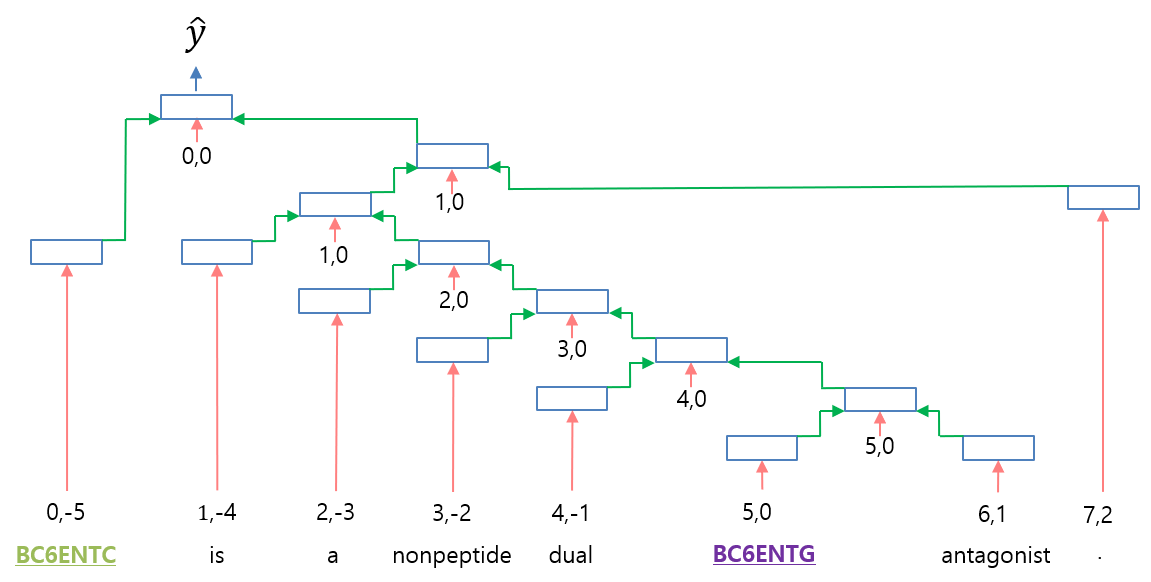


Supplementary Figure 2 Tree structure example for position feature. Each node has position feature of its own below the node.

Position Feature Generation

Position feature embedding represents the relative distance from each word position in a sentence to target entities. As we explained in the paper, the word “dual” is the fourth word that follows the first target entity and it is located right before the second target entity. Therefore, the node of the word “dual” has the position feature [4, -1]. We decided other leaf nodes in the same way and this is illustrated in Supplementary Figure 2. Every parent node in the tree structure has two child nodes. We transferred the smallest absolute position feature value from the child nodes to the parent node.

When we transferred the position features to the tree-LSTM model, we faced a technical issue in transferring negative values to the model. To tackle the issue, we added 9 to each distance value after the distance was determined.

**Input data format and data flow from a node’s perspective**

Input data format before the tree-LSTM model

After the subtree containment and position features are generated, the data for the tree-LSTM model has the format shown in the following example:

| **Example:** | (4/1/9/9 (4/1/9/4 BC6ENTC) (4/1/10/9 (4/1/10/9 (4/0/10/5 is) (4/1/11/9 (4/0/11/6 a) (4/1/12/9 (4/0/12/7 nonpeptide) (4/1/13/9 (4/0/13/8 dual) (4/1/14/9 (4/1/14/9 BC6ENTG) (4/0/18/10 antagonist)))))) (4/0/18/11 .))) |
| --- | --- |
| **Format:** | (label/Subtree Containment/Position1/Position2 content) |

The label is the answer label of an entity pair, and all the nodes of a tree share the same label. In the example above, the label 4 denotes that the instance has the label for the class “antagonist.” Subtree Containment, Position1, and Position2 are the features of a node. A node in a tree could be a leaf or an internal node. The content in a node is an input word if it is a leaf node, and if the current node is not a leaf node, the content consists of two child nodes of the current node.

Data flow from a node’s perspective

We explain how data is transferred from one node to another from a node’s perspective. From the above input data format example, we will focus on the following nodes only:

(4/1/14/9 (4/1/14/9 BC6ENTG) (4/0/18/10 antagonist))

The nodes can be illustrated as Supplementary Figure 3.


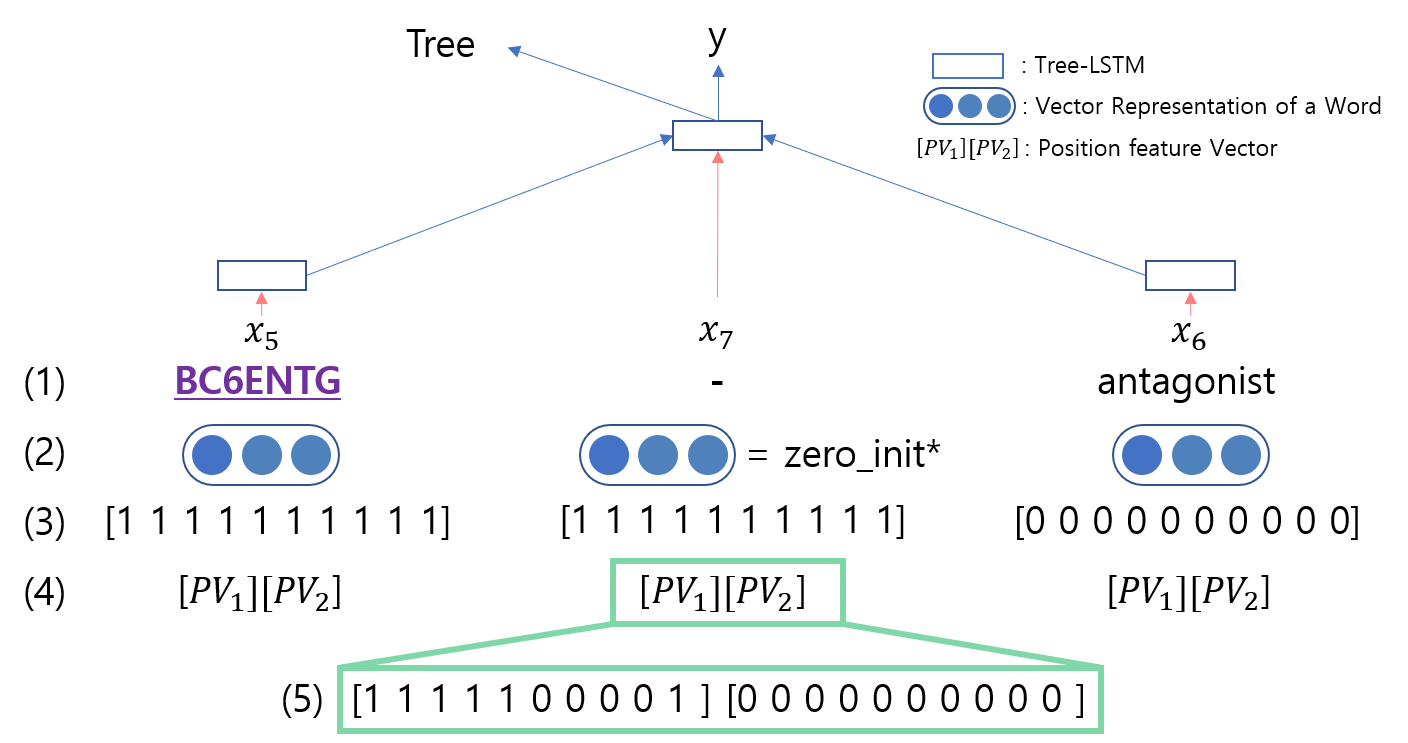


Supplementary Figure 3. Every node in the tree-LSTM model receives as input the two output vectors from the two child nodes and the input vector corresponding to the current node. (1) A word level content (2) Vector representations of words from a pre-trained word embedding. If a node is not a leaf, this vector representation is denoted as zeros. (3) Vector from subtree containment features. If the feature is 1, its vector elements are denoted as ones. (4) Position feature vectors. The position feature vectors inside the green box are from the position feature [5,0] (5) An example of the position feature vectors. Each position feature vector is from the smallest absolute number of the child nodes.

As shown in Figure 3, the vector $\boldsymbol{x}_{\boldsymbol{7}}$ consists of a subtree containment feature vector, position feature vector and the vector representation of a word in a sentence. If a node is not a leaf, the vector representation of the node is denoted as zero. The size of the input vector corresponding to $\boldsymbol{x}_{\boldsymbol{7}}$ is 230 (10 + 20 + 200). Every tree-LSTM node receives as input the outputs of the two child nodes and the input vector corresponding to the current node.

| $\tilde{h_{j}}= \sum_{k\in B\left( j \right)} h_{k},$ | (1) |
| --- | --- |

The input values $\boldsymbol{h}_{\boldsymbol{k}}$ from the set of child nodes B(j) can be calculated using Equation (1) where subscript k means that $\boldsymbol{h}_{\boldsymbol{k}}$ is from the k-th child node. For any node j, we have the following tree-LSTM equations:

| $i_{j}= \sigma(W^{i}\left[ x_{j},\tilde{h_{j}},e_{j} \right]+ b^{i})$ | (2) |
| --- | --- |
| $f_{jk}= \sigma(W^{f}\left[ x_{j},h_{k},e_{j} \right]+ b^{f})$ | (3) |
| $o_{j}= \sigma(W^{o}\left[ x_{j},\tilde{h_{j}},e_{j} \right]+ b^{o})$ | (4) |
| $u_{j}= tanh(W^{u}\left[ x_{j},\tilde{h_{j}},e_{j} \right]+ b^{u})$ | (5) |
| $c_{j}= i_{j}\odot drop(u_{j})+ \sum_{k\in B(j)} f_{jk}\odot c_{k}$ | (6) |
| $h_{j}= o_{j}\odot tanh(c_{j})$ | (7) |
| $\mathrm{drop}\left( x \right)= \left\{ \begin{aligned} mask*x, if train phase, \\ x otherwise \end{aligned} \right.$ | (8) |

In Equations (2-7), $x_{j}$ is the whole input vector and i, f, o, c, and h are the input gate, forget gate, output gate, memory cell, and the hidden state, respectively. $e_{j}$ is the result vector of tracking LSTM which is required for SPINN. Since the tree-LSTM model does not use this vector, the vector is denoted as zeros for the tree-LSTM model. $u_{j}$is a temporary vector used in the computation of the memory cell state. Equations (2-5), in which drop(x) is a recurrent dropout function, describe the fully connected layer.

After the process, a tree-LSTM node has two output values: $h_{j}$ and $c_{j}$. The output values are transferred to the parent node and the parent tree-LSTM node calculates the values like its child node. We can predict the instance class using the $h_{j}$ based on two leaf nodes; however, we do not predict the class label until the node is the root node.

| $\tilde{p}\left( y \vert x_{j} \right)= W^{{(f}_{c})}h_{j}+ b^{{(f}_{c})}$ | (9) |
| --- | --- |
| $\tilde{y}= argmax \tilde{p}\left( y \vert x_{j} \right)$ | (10) |

When the node j is the root node, we compute the fully connected layer for output. Equation (9) is the fully connected layer and the output size of Equation (10) is the number of class groups. We choose the predicted value y for an output.

| $J\left( \theta\right)=-\frac{1}{m}\sum_{k}^{m} y^{k}log(softmax\left( \tilde{p}\left( y^{k} \vert x^{k} \right) \right))$ | (11) |
| --- | --- |

After the final logit score is calculated, we use the softmax cross-entropy classifier as the cost function. m is the total number of items in the training set.

**Ensemble Method**


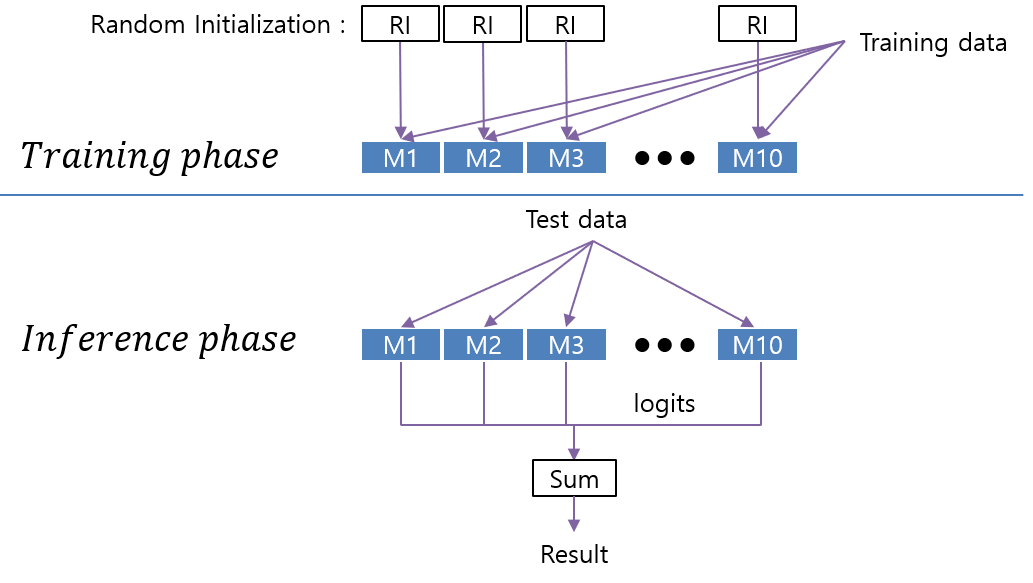


Supplementary Figure 4 RI is the random initialization. M1 to M10 are the models that use same structure and hyperparameter. We sum the output probabilities (logits) of ensemble members, which are generated using the same neural network model with random weight initialization.

We illustrate the ensemble method in Supplementary Figure 4. We consider the models used in the ensemble method as ensemble members. The ensemble members are generated using the same neural network model with random weight initialization. We use the ensemble method to boost the performance of the ensemble members that have the same structure and use the same hyperparameter. In the training phase, we independently trained each ensemble member with random weight vector initialization. When inferring a relation in an easy sample, the trained ensemble members make relatively consistent predictions, but when inferring from a difficult sample, the trained ensemble members may make slightly different predictions. We use the weighted voting of 10 ensemble members to produce robust results.

In summary, the prediction results of ambiguous samples can vary depending on how the model is trained. We resolve this problem to some extent by reducing the variance of our model, and thus achieve robust performance using the ensemble method.
